# Application of artificial intelligence in endoscopic image analysis for the diagnosis of a gastric cancer pathogen-*Helicobacter pylori* infection

**DOI:** 10.1038/s41598-023-40179-5

**Published:** 2023-08-17

**Authors:** Chih-Hsueh Lin, Ping-I Hsu, Chin-Dar Tseng, Pei-Ju Chao, I-Ting Wu, Supratip Ghose, Chih-An Shih, Shen-Hao Lee, Jia-Hong Ren, Chang-Bih Shie, Tsair-Fwu Lee

**Affiliations:** 1 Department of Electronic Engineering, National Kaohsiung University of Science and Technology, Kaohsiung, 80778 Taiwan; 2 Medical Physics and Informatics Laboratory of Electronic Engineering, National Kaohsiung University of Science and Technology, Kaohsiung, 80778 Taiwan; 3 Division of Gastroenterology, Department of Medicine, An Nan Hospital, China Medical University, Tainan, Taiwan; 4 Department of Education and Research, An Nan Hospital, China Medical University, Tainan, Taiwan; 5 Division of Gastroenterology and Hepatology, Department of Internal Medicine, Antai Medical Care Corporation, Antai Tian-Sheng Memorial Hospital, Donggan, Pingtung County Taiwan; 6 Department of Nursing, Meiho University, Neipu, Pingtung County Taiwan; 7 Department of Radiation Oncology, Linkou Chang Gung Memorial Hospital and Chang Gung University College of Medicine, Linkou, Taiwan; 8 Department of Medical Imaging and Radiological Sciences, Kaohsiung Medical University, Kaohsiung, 80708 Taiwan; 9 PhD Program in Biomedical Engineering, Kaohsiung Medical University, Kaohsiung, 80708 Taiwan; 10 School of Dentistry, College of Dental Medicine, Kaohsiung Medical University, Kaohsiung, 80708 Taiwan

**Keywords:** Biological techniques, Biophysics, Cancer, Diseases

## Abstract

*Helicobacter pylori* (*H. pylori*) infection is the principal cause of chronic gastritis, gastric ulcers, duodenal ulcers, and gastric cancer. In clinical practice, diagnosis of *H. pylori* infection by a gastroenterologists’ impression of endoscopic images is inaccurate and cannot be used for the management of gastrointestinal diseases. The aim of this study was to develop an artificial intelligence classification system for the diagnosis of *H. pylori* infection by pre-processing endoscopic images and machine learning methods. Endoscopic images of the gastric body and antrum from 302 patients receiving endoscopy with confirmation of *H. pylori* status by a rapid urease test at An Nan Hospital were obtained for the derivation and validation of an artificial intelligence classification system. The *H. pylori* status was interpreted as positive or negative by Convolutional Neural Network (CNN) and Concurrent Spatial and Channel Squeeze and Excitation (scSE) network, combined with different classification models for deep learning of gastric images. The comprehensive assessment for *H. pylori* status by scSE-CatBoost classification models for both body and antrum images from same patients achieved an accuracy of 0.90, sensitivity of 1.00, specificity of 0.81, positive predictive value of 0.82, negative predicted value of 1.00, and area under the curve of 0.88. The data suggest that an artificial intelligence classification model using scSE-CatBoost deep learning for gastric endoscopic images can distinguish *H. pylori* status with good performance and is useful for the survey or diagnosis of *H. pylori* infection in clinical practice.

## Introduction

*Helicobacter pylori* (*H. pylori*) infects the epithelial lining of the stomach and is the major cause of chronic gastritis, peptic ulcer disease, and gastric cancer^1^. *H. pylori* eradication has become the standard therapy to cure peptic ulcer disease^1^. In regions with a high incidence of gastric adenocarcinoma, eradication of *H. pylori* is advocated to prevent the development of gastric cancer^2^.

Several diagnostic methods utilizing invasive or non-invasive techniques with varying levels of sensitivity and specificity have been developed to detect *H. pylori* infection. Invasive methods including rapid urease test, histology, and culture require endoscopy with biopsies of gastric tissues^3^. Rapid urease test is based on the production of urease enzyme by *H. pylori* bacteria. The sensitivity the test are significantly lower in patients with intestinal metaplasia and also in the cases with peptic ulcer bleeding^4–6^. Additionally, treatment with proton-pump inhibitors, antibiotics, and bismuth compounds may also lead to false-negative results because these agents can prevent the production of urease by *H. pylori*^3^. Furthermore, several organisms such as *Klebsiella pneumoniae*, *Staphylococcus aureus*, *Proteus mirabilis*, *Enterobacter cloacae*, and *Citrobacter freundii* in the oral cavity or stomach also present urease activity and may give false-positive results^6^. Histology is more expensive than rapid urease test. Many factors affect the diagnostic accuracy of histological examination, such as the number and location of the collected biopsy materials, the experiences of pathologists, the staining techniques, PPIs or antibiotic use, and the presence of other bacterial species^4^, but with structural similarity to *Helicobacter*^7^.

Several studies have demonstrated that the judgment of *H. pylori* infection by conventional white light endoscopy could be based on the presence of diffuse redness, rugal hypertrophy, or thick and whitish mucus^8^. However, diagnosis by the impression of a gastroenterologist using endoscopic images is inaccurate and cannot be used for the management of gastrointestinal diseases in clinical practice^8^.

Recently, emerging studies have highlighted the application of artificial intelligence in the diagnosis of gastrointestinal diseases^9–11^. For example, the application of deep learning to endoscopic images by a Convolutional Neural Networks (CNN) has been used to detect small intestine or colon lesions^12^ and to assess the invasion depth of gastric cancer^13–15^. Deep learning with the computer-aided analysis of endoscopic images using CNN has also been developed for the diagnosis of *H. pylori* infection^10,16,17^. However, several studies applying artificial intelligence in the diagnosis of *H. pylori* infection used inadequate tests as gold standards for diagnosis such as serum *H. pylori* antibody^10,16^ and urine *H. pylori* antibody^17^. In fact, a positive test of serum or urine *H. pylori* antibody indicates the tested subjects with either active or past *H. pylori* infection. Therefore, these studies using antibody tests as gold standards for active *H. pylori* infection might have a false-positive result for those with past *H. pylori* infection, and the inadequate gold standard would impair the diagnostic accuracy of developed AI system for *H. pylori* diagnosis. Additionally, some of these studies excluded patients with peptic ulcer and gastric cancer from the investigated population^18^. Exclusion of these important target populations might limit the generalizability of the CNN decision system for the diagnosis of *H. pylori* infection.

With regard to the artificial intelligence technology in the diagnosis of gastrointestinal diseases, Liu et al. proposed two sub-networks: O-stream and P-stream. The original image was used as an O-stream. The input extract color, global features, and preprocessed image were used as the input of the P-stream to extract texture and detailed features^19^. Sobri et al. proposed a computer visualization technology to extract features from texture and color and to extract features of the Gray-Level Co-occurrence Matrix (GLCM) from the wavelet transformed image. They used discrete wavelet transform on the endoscopic image, classified the endoscopic gastritis image with image features, and then combined the texture and color moment features to develop a classifier model, SVM^20^. Many pre-processing articles use discrete wavelet transform, GLCM, and color space conversion methods to extract texture features.

Hierarchical feature engineering in high dimensional learned kernels from the complex connection of parameters and nonlinear activation function makes the learned features in the CNN augur well, with the benefit of translation invariance. However, many methods in the past used separate modules with deep learning to extract features of images concerned with the nature of the underlying problem. Jain et al. proposed a CNN-based WCENet model for anomaly detection and positioning in Wireless Capsule Endoscopy (WCE) images^21^. Zhang et al. proposed a dense CNN network-based stereo matching method with multiscale feature connections as Dense-CNN. A new dense connection network with multiscale convolutional layers was constructed using Dense-CNN. The rich image features were extracted, and the combined multiscale features with context information were used to estimate the cost of stereo matching. The experimental results with the proposed new loss function strategy have been used to learn neural network parameters more reasonably, which can improve the performance of the proposed Dense-CNN model in disparity calculation^22^. Several previous studies have shown that a cognitive visual attention mechanism that adds to the CNN network architecture can extract more important features from the original image and improve the performance of artificial intelligence.

Currently, diagnosis of *H. pylori* infection during endoscopy requires gastric biopsies with rapid urease test, histology or culture in clinical practice. However, gastric biopsies with aforementioned tests require biopsy instruments and costs of rapid urease test, histology and culture. Additionally, histological examination and culture of *H. pylori* are time-consuming. Furthermore, gastric biopsy may induce bleeding in patients taking antiplatelet or anticoagulant agents and those with coagulopathy. If a novel artificial intelligence system using real-time endoscopic images has a similar ore even higher diagnostic accuracy for *H. pylori* infection as aforementioned biopsy methods, it may replace these diagnostic modalities and also can save medical cost, provide immediate diagnosis and avoid biopsy-induced bleeding in patients with bleeding tendency.

In this study, we hypothesized that artificial intelligence learning technology can accurately assess *H. pylori* status by endoscopic images, and aimed to develop a novel artificial intelligence classification system for the diagnosis of *H. pylori* infection by CNN and Concurrent Spatial and Channel Squeeze and Excitation (scSE) network, combined with different classification models for deep learning of gastric images. In order to increase the generalizability of the artificial intelligence classification system, we included the subjects with and without major upper gastrointestinal diseases such as peptic ulcer and gastric cancer. In addition, we used an accurate method, rapid urease test, as the gold standard for the diagnosis of *H. pylori* infection in this study. Furthermore, the current study used the CNN model and the attention technology, which could improve the body and antrum images with a better classification effect.

## Materials and methods

### Patient population

Patients receiving endoscopy with gastric biopsies for rapid urease test at An Nan Hospital (Tainan, Taiwan) from October 2020 to December 2021 were retrospectively searched. The exclusion criteria included (1) previous eradication treatment for *H. pylori* infection, (2) history of gastrectomy, (3) use of antibiotics within the previous 4 weeks, (4) use of proton pump inhibitor within 2 weeks before endoscopy (5) coexistence of serious concomitant illness (for example, decompensated liver cirrhosis, uremia, and malignancy), and (6) upper gastrointestinal bleeding. The patients were divided into 5 equal subsets, and each subset had about 60 patients. The endoscopic images from the first three subsets of patients (n = 182) receiving endoscopy between October 2020 and June 2021 were assigned to the derivation group for creating an artificial intelligence classification system in the diagnosis of *H. pylori* infection. The endoscopic images from the other two subsets of patients (n = 120) receiving endoscopy between July 2021 and December 2021 were assigned to the validation group for assessing the accuracy of the derived artificial intelligence classification system. The study protocol was approved by the Institutional Review Board of the An Nan Hospital of China Medical University (TMANH109-REC008). The Institutional Review Board waived informed consent requirement of the study because it was a retrospective work.

### Upper endoscopy and gastric images

Upper endoscopy was performed using a standard endoscope (GIF-Q260J; Olympus, Tokyo, Japan). Gastric images captured during high-definition, white-light examination of the antrum (forward) and body (forward and retroflex) were used for both the derivation and validation datasets. An antral gastric biopsy specimen and a body biopsy specimen were obtained for rapid urease test. *H. pylori* status was determined by the results of rapid urease test (Delta West Bentley, WA, Australia)^23^. Archived gastric images obtained during standard white-light examination from the endoscopic database were extracted. Two endoscopists independently screened and excluded images that were suboptimal in quality (i.e., blurred images, excessive mucus, food residue, bleeding, and/or insufficient air insufflation). The representative areas were then independently selected by two endoscopists according to standard selection criteria. The standard criteria for representative image selection included (1) clear images, (2) no bubbles, blood or food residue, (3) no reflex light, and (4) no specific lesions (e.g., erosion, ulcer or tumor). No special tool was used for representative area selection. Table 1 shows the numbers of patients and images in the derivation and validation groups. The major gastrointestinal diseases that patients suffered from included gastroesophageal reflux disease (n = 69), non-ulcer dyspepsia (n = 199), gastric ulcer (n = 20), duodenal ulcer (n = 12), and gastric cancer (n = 2).

**Table 1:** Numbers of patients and images in the derivation and validation datasets.

*H. pylori* infection status	No. of patients	No. of images
Derivation dataset		
Positive	101	Antrum: 172 Body: 152
Negative	81	Antrum: 138 Body: 122
Validation dataset		
Positive	65	Antrum: 110 Body: 97
Negative	55	Antrum: 91 Body: 77

**Figure 1:**
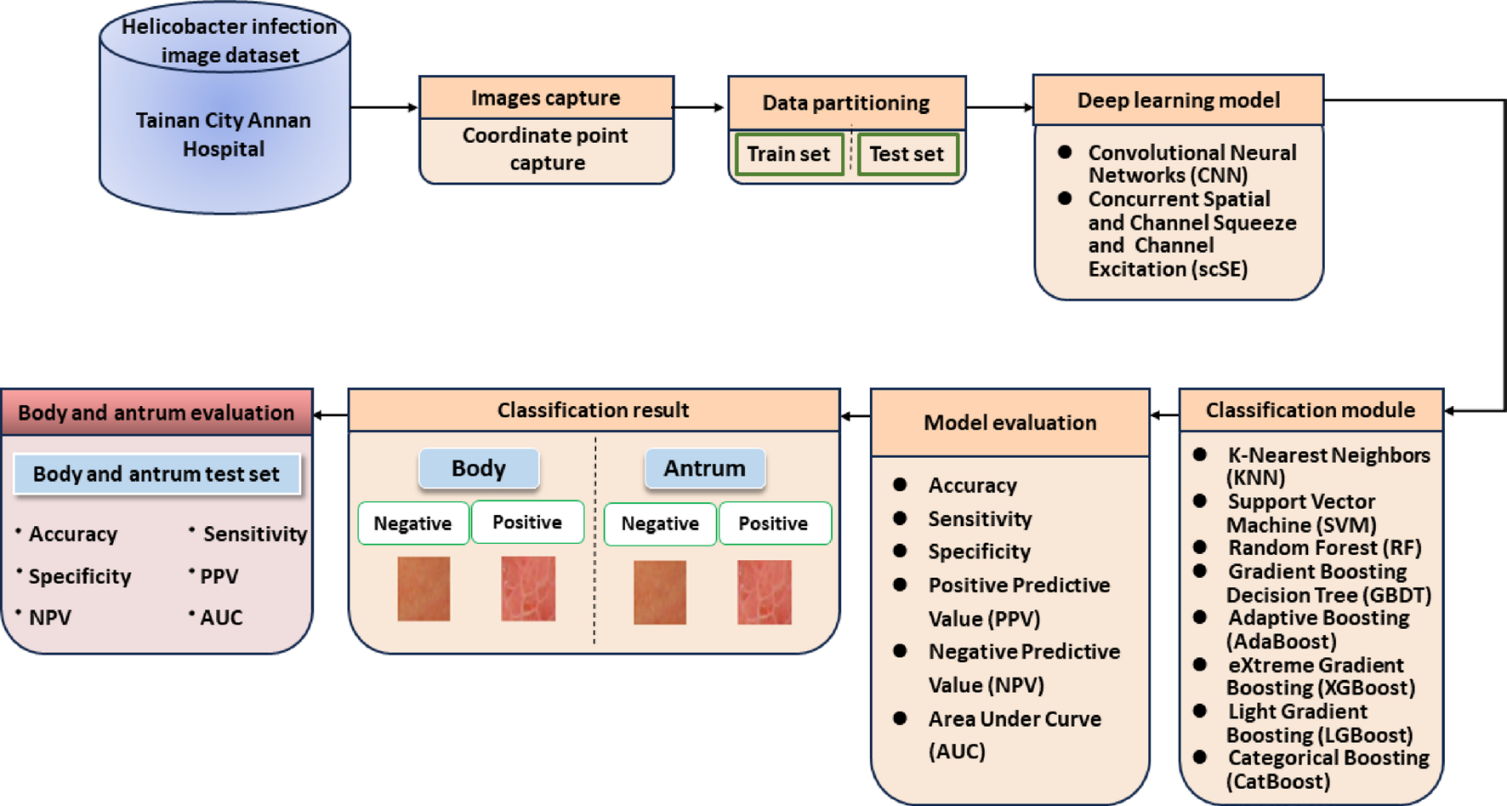
Overall research flow chart.

**Figure 2:**
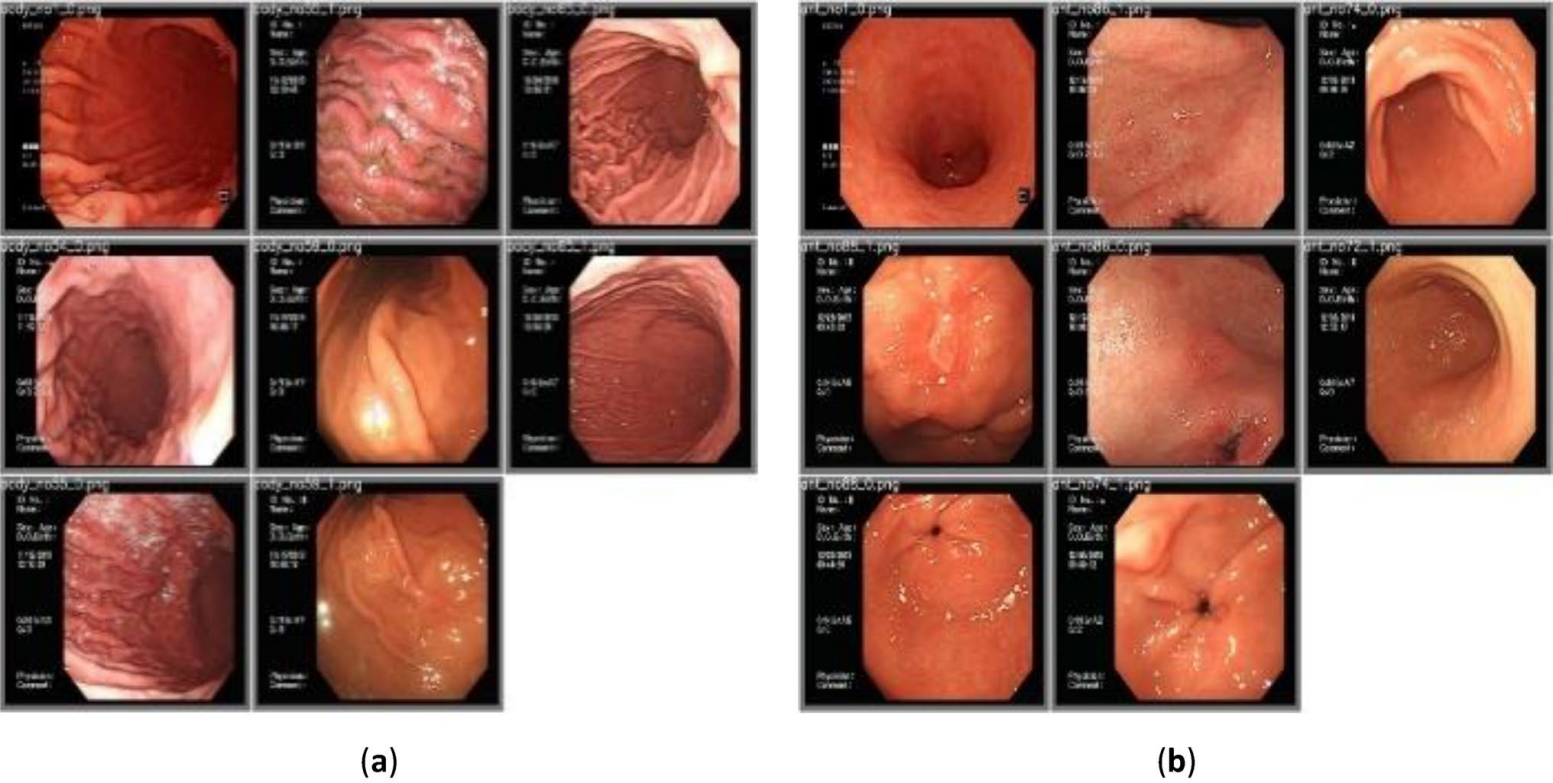
(**a**) Endoscopic images of the body and (**b**) Endoscopic images of the antrum.

Figure 1 demonstrates the overall research flowchart. Endoscopic images of the gastric body and antrum from patients receiving endoscopy with confirmation of *H. pylori* status by rapid urease test were obtained for the derivation of an artificial intelligence classification system. The CNN and scSE network, combined with different classification models for deep learning of gastric images. The characteristics of the sample images were extracted effectively, and the classification model used the gastroscopic images from the antrum or body to make a comprehensive evaluation and diagnosis. All methods were performed in accordance with the relevant guidelines and regulations. The total number of patients providing endoscopic images was 302, of which 136 were *H. pylori*-negative and 166 were *H. pylori*-positive. Table 1 shows the numbers of patients and images in the derivation and validation datasets. The endoscopic images were obtained from the gastric antrum and body (Fig. 2). *H. pylori* status in the two gastric parts was classified into positive or negative categories using the artificial intelligence classification model.

### Capture image

**Figure 3:**
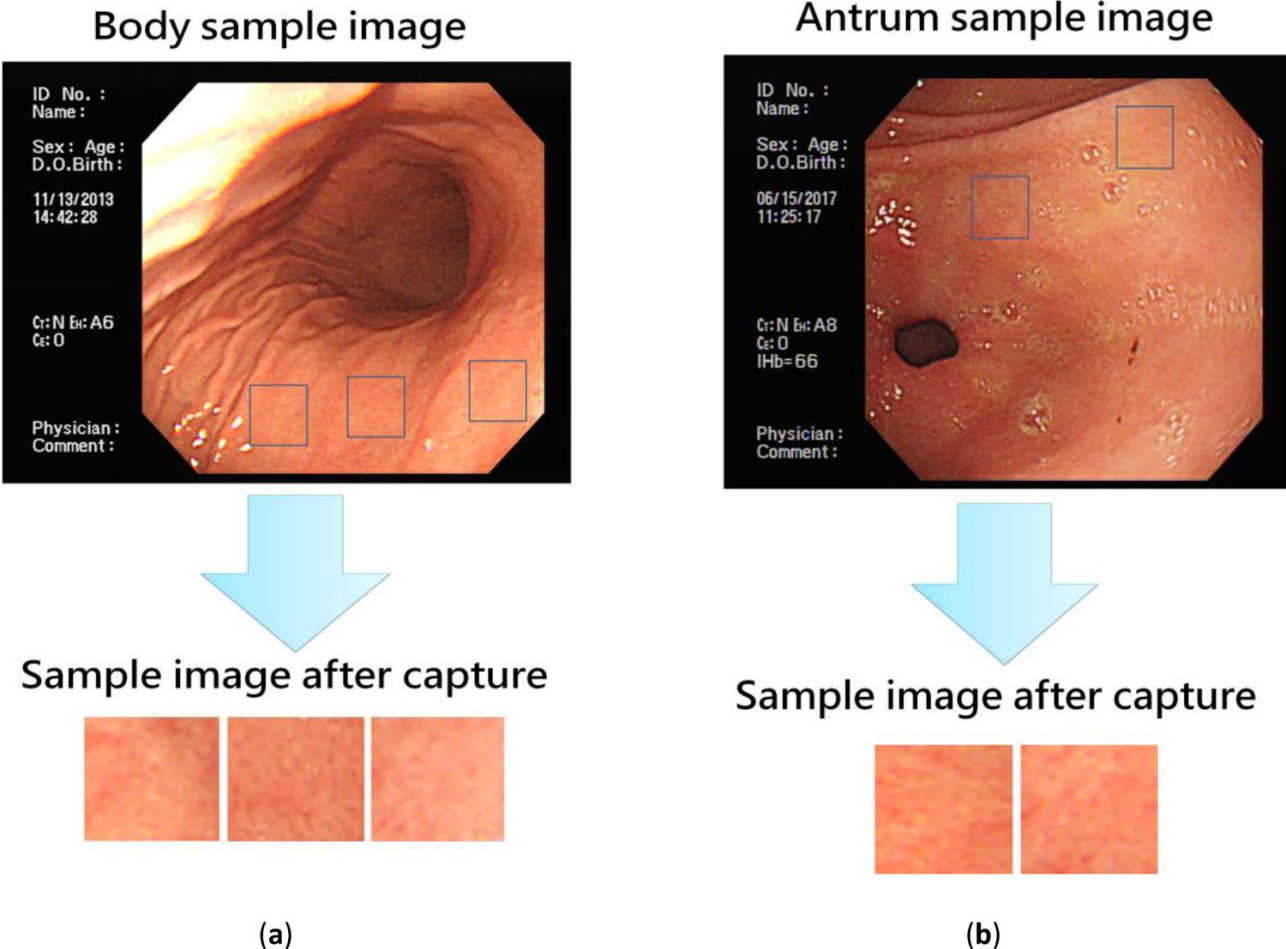
(**a**) Body and (**b**) Antrum image capture.

Since the original input image affected the accuracy of the output result, unnecessary feature information was removed. As shown in Fig. 3, the representative area was selected by the endoscopists for image capture. Because the traditional image pre-processing method may destroy the original important features of the image and cannot improve the accuracy of machine learning classification, we did not use any traditional image pre-processing technology in this study. Two deep learning neural network models, CNN and scSE, were directly used to extract the image features to facilitate subsequent analysis of image features by various machine learning classification methods.

### Convolutional neural networks (CNN)

The problem with traditional deep learning models is that they ignore three dimensional information, such as the horizontal, vertical, and color channels of the data. For CNN^24^, each image in the train and test set of images passes through a series of layers, including the convolutional, pooling, and fully connected layers. Among these, the convolutional and pooling layers can maintain shape characteristics to avoid a large increase in parameters, while the fully connected layer will be extracted. The image feature uses the connection between each neuron and the upper neuron to perform the final classification^25^. Because CNN has a shared weight architecture and translation invariance features, and feature extraction and classification can be generated at the same time during training, allowing the network to learn more effectively in parallel^26^, so it has excellent results in image data work^27^.

### Spatial and channel squeeze and excitation block (scSE)

**Figure 4:**
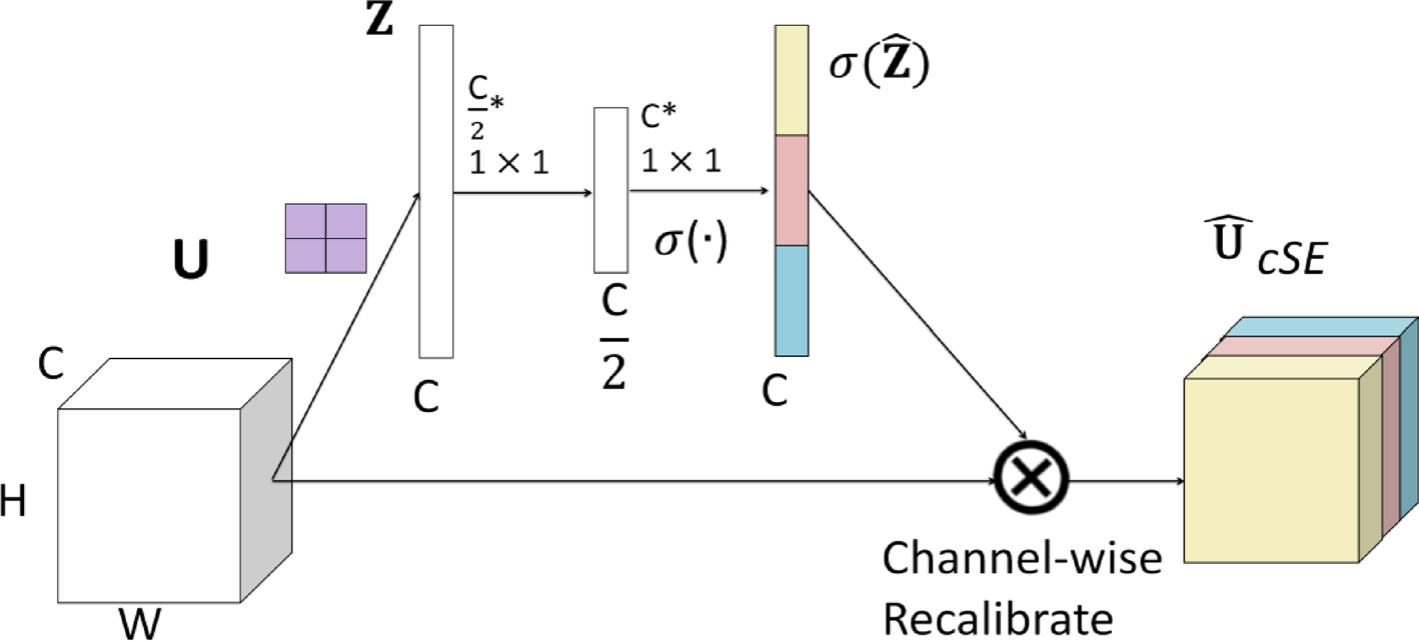
Channel attention mechanism of cSE architecture model.

**Figure 5:**
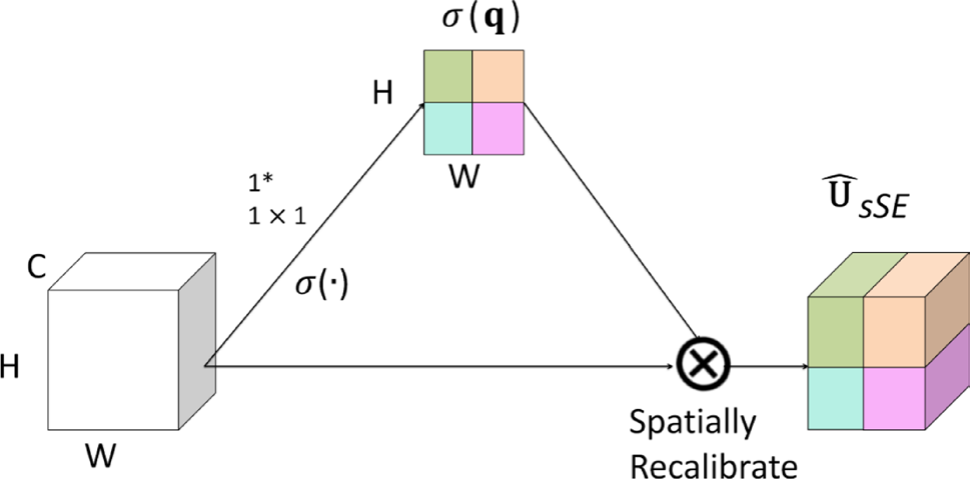
Spatial attention mechanism of sSE architecture model.

**Figure 6:**
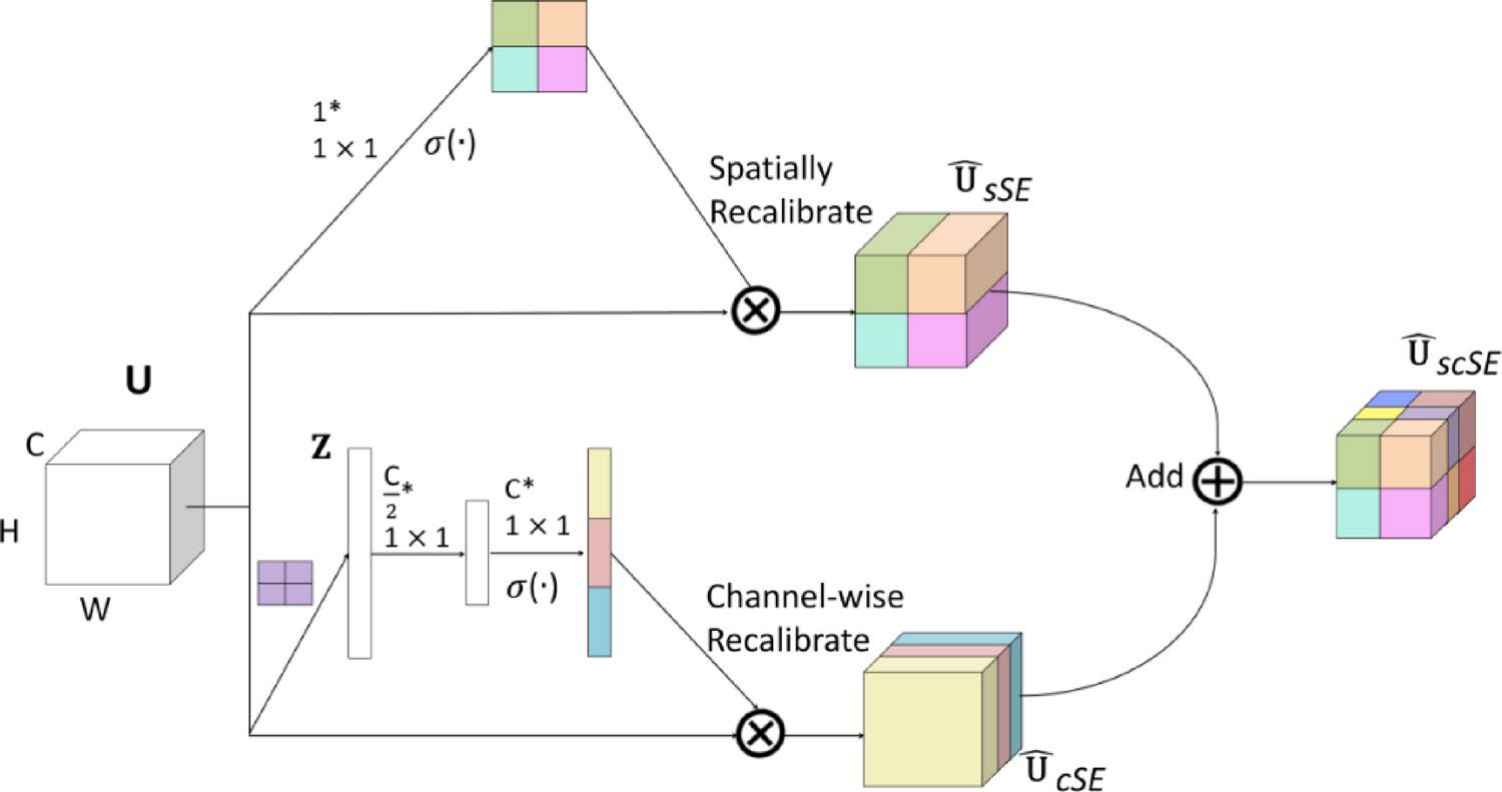
A scSE architecture model composed of cSE and sSE.

For the scSE network^28^, the Spatial Squeeze and Channel Excitation Block (cSE) and Channel Squeeze and Spatial Excitation Block (sSE) models were used to adjust the network features and were regarded as important effective feature maps or feature channels. Weight was used to weigh and reduce the impact of unimportant features. Therefore, useful information was given a higher weight, while invalid information was given a lower weight^29^. As shown in Fig. 4, in the cSE model, the C × W × H feature vector of the feature map was converted to C × 1 × 1 through global average pooling, and then two 1 × 1 × 1 feature information was used for processing to obtain C-dimensional feature information, normalized using the Sigmoid activation function, and finally multiplied channel wise to obtain the feature map of cSE^30^. As shown in Fig. 5, the sSE model was a spatial attention mechanism, which mainly used 1 × 1 convolution to compress the original feature map to form a change from C × W × H to 1 × W × H, and then the Sigmoid function layer normalized the feature information from 0 to 1 and obtained the feature map of spatial attention, and finally directly added it to the original feature map to complete the spatial information calibration^31^. As shown in Fig. 6, the scSE was mainly composed of a parallel connection of two modules, cSE and sSE. After the original feature map passed through the sSE and cSE models, two modules were added to obtain a more accurate and calibrated feature map^32^.

### Derivation and training algorithm

**Figure 7:**
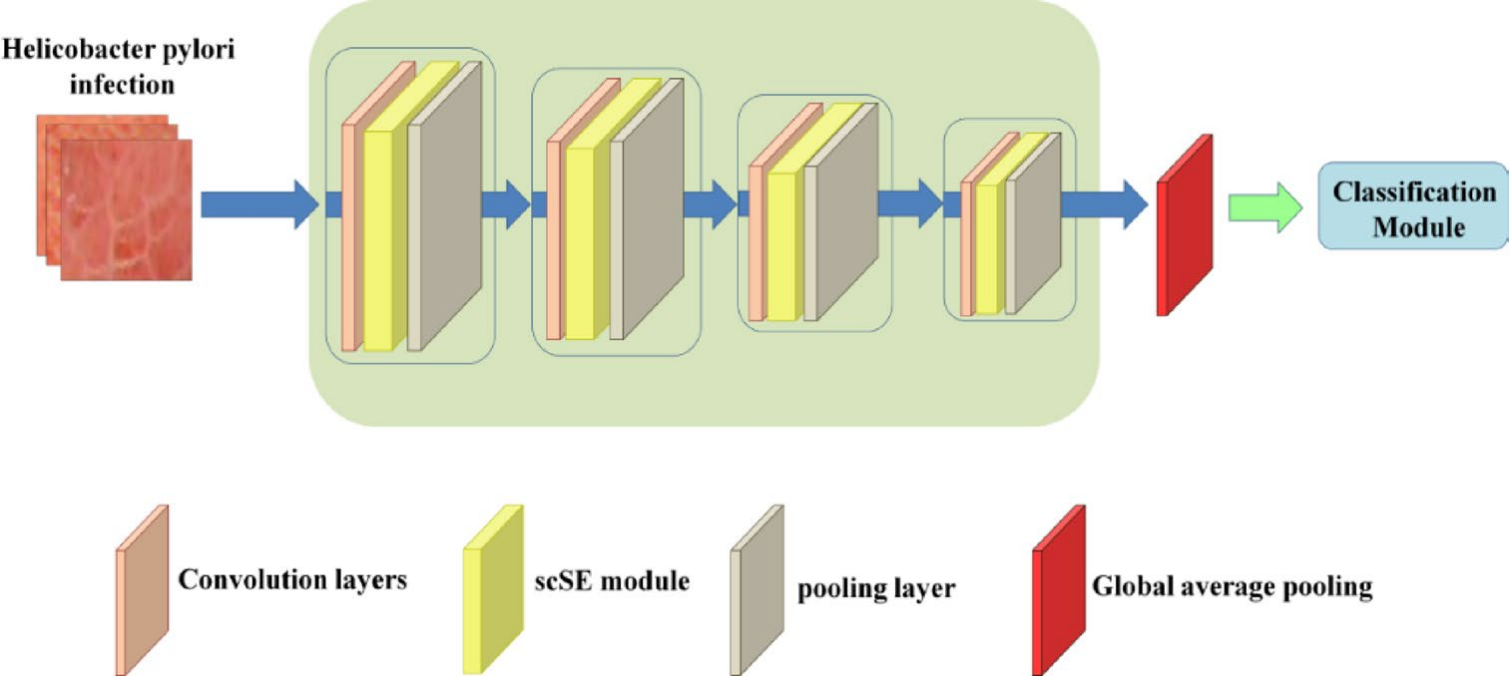
Convolutional network combined with classification model.

The endoscopic images of 182 patients were used for deep machine learning. Classification is the process of predicting the category of a given data point and belongs to the category of supervised learning, in which the target is also provided with input data^33–35^. For the need to predict *H. pylori* infection, it is more suitable to use classification algorithms for classification. As shown in Fig. 7, the layers of the feature extraction network were stacked from the original two to four, and finally, the last layer was used to match the input of the classification model such as KNN, SVM, RF, GBDT, AdaBoost, XGBoost, LGBoost, CatBoost. The output of the network was globally average pooled, and the original 8 × 8 × 256 dimensions were compressed to one dimensional data to allow the classification model to classify.

### Validation algorithm

Endoscopic images from 120 patients were used to evaluate the performance of the derived Artificial intelligence diagnostic system. There are different evaluation index methods for each machine learning model, and many evaluation indexes can be used to measure the performance of the classification model or prediction. The adjustment of parameters and feature selection of different models are typically used to achieve better evaluation performance and to help monitor and evaluate the situation to make appropriate fine tuning parameters and optimization goals^36^. In this study, six evaluation metrics were used to judge the performance of each classification model: accuracy, positive predictive value (PPV), negative predictive value (NPV), sensitivity, specificity, and area under the curve (AUC). Table 2 shows the confusion matrix for binary classification. The confusion matrix in predictive analysis was composed of true negative (TN, the predicted result was negative, and True was also negative), false negative (FN, predicted result was negative; however, the actual result was positive), false positive (FP, predicted result was positive, but the actual result was interpreted as negative), and true positive (TP, predicted result was positive, but the actual result was also positive)^37^. The performance of artificial intelligence diagnostic system for a single gastric image was assessed. Chi-square test was used to compare the performance of the different models. Differences were considered statistically significant at *P* < 0.05. Because the distribution of *H. pylori* on the surface of the stomach is heterogeneous through the gastric antrum and body, we also assessed the performance of the scSE-CatBoost diagnostic module for the representative images from the antrum and the body of the same patients.

**Table 2:** Confusion matrix for binary classification.

Predict result	Positive	Negative
Positive	TP	FP
Negative	FN	TN

*TP* true positive, *FP* false positive, *FN* false negative, *TN* true negative.

### Informed consent statement

All authors have confirmed the manuscript and approved the publication of the manuscript.

### Approval statement

A statement to confirm that all experimental protocols were approved by An Nan Hospital Medical Foundation Human Body Experiment Committee.

## Results

Each machine learning model can effectively help in understanding the performance of the model for the evaluation results. Therefore, this study used the attention mechanism and a combination of classification models to classify positive and negative and to evaluate and compare the two parts of the body and antrum infected by *H. pylori*. The classification methods of K-Nearest Neighbor (KNN)^38^, Support Vector Machine (SVM)^39^, Adaptive Boosting (AdaBoost)^40^, Random Forest (RF)^41^, Gradient Boosting Decision Tree (GBDT)^42^, eXtreme Gradient Boosting (XGBoost)^43^, Light Gradient Boosting (LGBoost)^44^, and Categorical Boosting (CatBoost)^45^ were used on the CNN and scSE models. The performance of each model was assessed by six parameters including accuracy, sensitivity, specificity, PPV, NPV, and AUC.

### Performance of CNN or scSE combined with different classification models for the diagnosis of *H. pylori* infection by endoscopic images from the gastric body or antrum

Table 3 shows the performance of CNN combined with different classification models for the diagnosis of *H. pylori* infection using endoscopic images from the gastric body. The CNN-CatBoost classification model had the best performance, with an accuracy of 88%, sensitivity of 93%, specificity of 80%, and AUC of 0.87. Table 4 displays the performance of scSE combined with different classification models for the diagnosis of *H. pylori* infection using endoscopic images from the gastric body. The scSE-LGBoost classification model achieved the best performance with an accuracy of 90%, sensitivity of 93%, specificity of 83%, and AUC of 0.88.

**Table 3:** Performance of CNN combined with different classification models for the diagnosis of *H. pylori* infection by single endoscopic image from gastric body.

Method	Accuracy	Sensitivity	Specificity	PPV	NPV	AUC
CNN	0.86	0.96	0.69	0.85	0.89	0.82
CNN-KNN	0.85	0.91	0.73	0.86	0.82	0.82
CNN-SVM	0.84	0.91	0.70	0.85	0.81	0.81
CNN-RF	0.87	0.93	0.77	0.88	0.85	0.85
CNN-GBDT	0.82	0.88	0.72	0.85	0.76^a^	0.80
CNN-AdaBoost	0.87	0.92	0.77	0.88	0.84	0.84
CNN-XGBoost	0.88	0.93	0.79	0.89	0.86	0.86
CNN-LGBoost	0.88	0.92	0.79	0.89	0.85	0.86
CNN-CatBoost	0.88	0.93	0.80	0.90	0.86	0.87

CNN, KNN, SVM, RF, GBDT, AdaBoost, XGBoost, LGBoost, CatBoost, PPV, NPV and AUC are short for Convolutional Neural Networks, K-Nearest Neighbor, Support Vector Machine, Random Forest, Gradient Boosting Decision Tree, Adaptive Boosting, eXtreme Gradient Boosting, Light Gradient Boosting, Categorical Boosting, Positive Predictive Value, Negative Predictive Value and Area Under the ROC curve, ^a^*P* = 0.001 for CNN-GBDT vs CNN.

**Table 4:** Performance of scSE combined with different classification models for the diagnosis of *H. pylori* infection by single endoscopic image from gastric body.

Method	Accuracy	Sensitivity	Specificity	PPV	NPV	AUC
scSE	0.88	0.93	0.80	0.89	0.86	0.86
scSE-KNN	0.89	0.93	0.81	0.90	0.87	0.87
scSE-SVM	0.83	0.90	0.71	0.85	0.80	0.81
scSE-RF	0.88	0.94	0.76	0.88	0.88	0.85
scSE-GBDT	0.84	0.91	0.72	0.86	0.81	0.82
scSE-AdaBoost	0.87	0.90	0.81	0.90	0.82	0.86
scSE-XGBoost	0.89	0.93	0.80	0.90	0.86	0.87
scSE-LGBoost	0.90	0.93	0.83	0.91	0.87	0.88
scSE-CatBoost	0.88	0.93	0.80	0.89	0.86	0.86

scSE, KNN, SVM, RF, GBDT, AdaBoost, XGBoost, LGBoost, CatBoost, PPV, NPV and AUC are short for Spatial Squeeze and Channel Excitation Block, K-Nearest Neighbor, Support Vector Machine, Random Forest, Gradient Boosting Decision Tree, Adaptive Boosting, eXtreme Gradient Boosting, Light Gradient Boosting, Categorical Boosting, Positive Predictive Value, Negative Predictive Value and Area Under the ROC curve. No differences in the performances of accuracy, sensitivity, specificity, PPV, NPV and AUC between groups.

Table 5 lists the performance of CNN combined with different classification models for the diagnosis of H. pylori infection using endoscopic images from the gastric antrum. CNN-LGBoost had the best performance, with an accuracy of 87%, sensitivity of 89%, specificity of 86%, and AUC of 0.87. Table 6 demonstrates the performance of scSE combined with different classification models for the diagnosis of *H. pylori* infection by endoscopic images of the gastric antrum. Both scSE-KNN and scSE-CatBoost achieved the best performance, with an accuracy of 89%, sensitivity of 90%, specificity of 88%, and AUC of 0.89.

**Table 5:** Performance of CNN combined with different classification models for the diagnosis of *H. pylori* infection by single endoscopic image from gastric antrum.

Method	Accuracy	Sensitivity	Specificity	PPV	NPV	AUC
CNN	0.80	0.93	0.66	0.77	0.88	0.79
CNN-KNN	0.84	0.88	0.80^a^	0.84	0.84	0.84
CNN-SVM	0.83	0.86	0.78^b^	0.83	0.82	0.82
CNN-RF	0.83	0.85	0.81^c^	0.85	0.82	0.83
CNN-GBDT	0.78	0.82	0.73	0.78	0.77	0.77
CNN-AdaBoost	0.83	0.87	0.79^d^	0.83	0.83	0.83
CNN-XGBoost	0.87	0.88	0.86^e^	0.89	0.86	0.87
CNN-LGBoost	0.87	0.89	0.86^f^	0.89	0.86	0.87
CNN-CatBoost	0.86	0.89	0.84^g^	0.87	0.86	0.86

CNN, KNN, SVM, RF, GBDT, AdaBoost, XGBoost, LGBoost, CatBoost, PPV, NPV and AUC are short for Convolutional Neural Networks, K-Nearest Neighbor, Support Vector Machine, Random Forest, Gradient Boosting Decision Tree, Adaptive Boosting, eXtreme Gradient Boosting, Light Gradient Boosting, Categorical Boosting, Positive Predictive Value, Negative Predictive Value and Area Under the ROC curve. ^a^*P* = 0.002 for CNN-KNN vs CNN; ^b^*P* = 0.008 for CNN-SVM vs CNN; ^c^*P* = 0.001 for CNN-RF vs CNN; ^d^*P* = 0.004 for CNN-AdaBoost vs CNN; ^e^*P* < 0.001 for CNN-XGBoost vs CNN; ^f^*P* < 0.001 for CNN-LGBoost vs CNN; *P* < 0.001 for CNN-CatBoost vs CNN.

**Table 6:** Performance of scSE combined with different classification models for the diagnosis of *H. pylori* infection by single endoscopic image from gastric antrum.

Method	Accuracy	Sensitivity	Specificity	PPV	NPV	AUC
scSE	0.82	0.95	0.65	0.77	0.92	0.80
scSE-KNN	0.89	0.90	0.88^a^	0.90^i^	0.88	0.89
scSE-SVM	0.85	0.88	0.82^b^	0.86^k^	0.84	0.85
scSE-RF	0.86	0.87	0.84^c^	0.87^l^	0.84	0.86
scSE-GBDT	0.81	0.83	0.79^d^	0.83	0.80	0.81
scSE-AdaBoost	0.86	0.86	0.85^e^	0.87^m^	0.84	0.86
scSE-XGBoost	0.88	0.90	0.85^f^	0.88^n^	0.88	0.88
scSE-LGBoost	0.88	0.89	0.87^g^	0.90^o^	0.87	0.88
scSE-CatBoost	0.89	0.90	0.88^h^	0.90^p^	0.88	0.89

scSE, KNN, SVM, RF, GBDT, AdaBoost, XGBoost, LGBoost, CatBoost, PPV, NPV and AUC are short for Spatial Squeeze and Channel Excitation Block, K-Nearest Neighbor, Support Vector Machine, Random Forest, Gradient Boosting Decision Tree, Adaptive Boosting, eXtreme Gradient Boosting, Light Gradient Boosting, Categorical Boosting, Positive Predictive Value, Negative Predictive Value and Area Under the ROC curve. ^a^*P* < 0.001 for scSE-KNN vs scSE; ^b^*P* < 0.001 for scSE-SVM vs scSE; ^c^*P* < 0.001 for scSE-RF vs scSE; ^d^*P* = 0.002 for scSE-GBDT vs scSE; ^e^*P* < 0.001 for scSE-AdaBoost vs scSE; ^f^*P* < 0.001 for scSE-XGdaBoost vs scSE; ^g^*P* < 0.001 for scSE-LGdaBoost vs scSE; ^h^*P* < 0.001 for scSE-CatBoost vs scSE; ^i^*P* < 0.001 for scSE-KNN vs scSE; ^k^*P* = 0.021 for scSE-SVM vs scSE; ^l^*P* = 0.009 for scSE-RF vs scSE; ^m^*P* = 0.009 for scSE-AdaBoost vs scSE; ^n^*P* = 0.004 for scSE-XGBoost vs scSE; ^o^*P* < 0.001 for scSE-LGBoost vs scSE; ^p^*P* < 0.001 for scSE-CatBoost vs scSE.

### Comprehensive assessment of *H. pylori* status by the scSE-CatBoost classification model with endoscopic images of both the body and antrum

Table 7 shows the results of the comprehensive assessment for *H. pylori* status by the scSE-CatBoost classification model with endoscopic images of both the body and the antrum of same patients. In this comprehensive classification model, *H. pylori* status was judged as a negative result if both body image and antrum image were classified as a negative result by the scSE-CatBoost classification model. If either the body or antrum image was classified as a positive result by the scSE-CatBoost classification model, the *H. pylori* status in the comprehensive assessment was judged as a positive result. The comprehensive assessment with the scSE-CatBoost classification model using endoscopic images from the antrum and body of same patients had good performance with an accuracy of 90%, sensitivity of 100%, specificity of 81%, and AUC of 0.88.

**Table 7:** Comprehensive assessment for *H. pylori* status by scSE-CatBoost classification models with endoscopic images from the antrum and body of same patients.

Method	Accuracy	Sensitivity	Specificity	PPV	NPV	AUC
scSE-CatBoost	0.90	1.00	0.81	0.82	1.00	0.88

scSE, CatBoost, PPV, NPV and AUC are short forms for Spatial Squeeze and Channel Excitation Block, Categorical Boosting, Positive Predictive Value, Negative Predictive Value and Area Under the ROC curve.

## Discussion

In this study, we developed a novel artificial intelligence classification system for the diagnosis of *H. pylori* infection by endoscopic images using the CNN and scSE networks and machine learning methods. The sensitivity, specificity, and accuracy for predicting *H. pylori* status by scSE-CatBoost classification model using endoscopic images from both the antrum and the body were 100%, 81%, and 90%, respectively. The results indicate that scSE-CatBoost classification model can achieve a high accuracy for the diagnosis of *H. pylori* infection with white light endoscopic images. It is important to note that the negative predictive value of our artificial intelligence-assisted *H. pylori* diagnosis system was 100%. The possibility of positive *H. pylori* status of the patients receiving endoscopy is extremely low if our image diagnosis system shows negative result of *H. pylori* status. Therefore, it is not necessary to further perform biopsy to check *H. pylori* status during endoscopy. The avoiding unnecessary biopsy for *H. pylori* testing has clinical implications because it can decrease medical cost, save endoscopy time and prevent biopsy-induced bleeding in patients with bleeding tendency. Currently, we still suggest the endoscopists to perform biopsy with rapid urease test or histology to confirm the diagnosis of *H. pylori* infection in patients with positive predictions by our artificial intelligence diagnostic system because the positive predictive value of our diagnostic system is suboptimal (82%). It is necessary to further confirm the diagnosis of *H. pylori* infection before the administration of eradication therapy. Nonetheless, the accuracy of our artificial intelligence diagnostic system may be further improved by deep learning of more endoscopic images and application of new learning technologies in the future. If no differences in the accuracies between our artificial intelligence diagnostic system and rapid urease test or histology exist, our image diagnostic system has a great potential to replace current biopsy-dependent methods for *H. pylori* testing.

The current study has several innovative improvements in the diagnosis of *H. pylori* infection by CNN and scSE networks. First, we examined the performances of CNN and scSE networks combined with different classification models for the diagnosis of *H. pylori* infection. The results showed that scSE-CatBoost classification models could achieve a very high accuracy for the diagnosis of *H. pylori* infection. Second, we assessed endoscopic images obtained from white light endoscopic system that was commonly used in daily practice in endoscopic unites. Some studies used blue laser or linked color images to develop image classification system for the diagnosis of *H. pylori* infection. These images are not ready to obtain in most endoscopic units. Third, some artificial intelligence diagnostic systems excluded the endoscopic images from patients with peptic ulcer and gastric cancer from the investigated population and limited the generalizability of their image diagnostic system in patients with important gastrointestinal diseases. In the current study, we included the subjects with and without major upper gastrointestinal diseases in the process of developing the artificial intelligence image diagnostic system. Therefore, our artificial intelligence classification system can be applied for the diagnosis of *H. pylori* infection in the patients with peptic ulcer and gastric cancer. In addition, some previous artificial intelligence diagnostic system used inadequate tests (serum of urine *H. pylori* antibody tests as gold standards for the diagnosis of *H. pylori* infection^10,16^. In the current study, we used rapid urease test as the gold standard for the diagnosis of *H. pylori* infection in this study. The rapid urease test is a reliable testing for *H. pylori* infection with a sensitivity of 90–95% and a specificity of 95–100%^3^.

This study used deep learning combined with classification models for datasets of endoscopic images from the gastric body and antrum. The evaluation of the model mainly uses CNN and scSE for evaluation and comparison. The experimental results showed that the use of scSE had a higher evaluation effect, either using gastric body or antrum images. The main reason is that the scSE model can perform weighting operations on information channels to enhance effective information and suppress invalid information. After adding the scSE model, it has more nonlinearity for the overall network, which can better fit the complex correlation between channels, not only increasing the effectiveness of extracting features but also greatly reducing the number of parameters and calculations.

Our data showed that the comprehensive assessment by scSE-CatBoost classification models with endoscopic images of both the body and antrum had a good performance in determining *H. pylori* status. The performance of for *H. pylori* status by scSE-CatBoost classification models could achieve an accuracy of 0.90, a sensitivity of 1.00, a specificity of 0.81, and an AUC of 0.88.

Our study has several limitations. First, the assessment of endoscopic images was not real-time. In clinical practice, it is important for real-time assessment of *H. pylori* infection during live endoscopy. Second, we only included patients without previous *H. pylori* eradication therapy. It remains unclear whether the artificial intelligence-assisted image diagnosis system can be applied for post-eradication assessment for *H. pylori* status. Third, this study was a retrospective work, our artificial intelligence-assisted image diagnosis system still require prospective validation in other populations.

## Conclusions

In clinical practice, the judgment of *H. pylori* infection by gastroenterologists’ impression of endoscopic images is often inaccurate. The comprehensive assessment of gastric endoscopic images by the scSE-CatBoost classification model and deep learning can achieve good performance in the determination of *H. pylori* status. The current study suggests that a machine learning based Image recognition system can be applied to distinguish *H. pylori* status and has great potential to be applied in the survey or diagnosis of *H. pylori* infection during endoscopy.

## Data Availability

The datasets generated and analysed during the current study are not publicly available due to privacy or ethical restrictions but are available from the corresponding author on reasonable request.

## References

[1] Abadi ATB, Kusters JG (2016). Management of *Helicobacter pylori* infections. BMC Gastroenterol..

[2] Liou J-M, Malfertheiner P, Lee Y-C, Sheu B-S, Sugano K, Cheng H-C, Yeoh K-G, Hsu P-I, Goh K-L, Mahachai V (2020). Screening and eradication of *Helicobacter pylori* for gastric cancer prevention: the Taipei global consensus. Gut.

[3] Sabbagh P, Mohammadnia-Afrouzi M, Javanian M, Babazadeh A, Koppolu V, Vasigala VR, Nouri HR, Ebrahimpour SJ (2019). Diagnostic methods for *Helicobacter pylori* infection: ideals, options, and limitations. Eur. J. Clin. Microbiol. Infect. Dis..

[4] Braden BJB (2012). Diagnosis of *Helicobacter pylori* infection. BMJ.

[5] Lewis JD, Kroser J, Bevan J, Furth EE, Metz DCJ (1997). Urease-based tests for Helicobacter pylori gastritis: Accurate for diagnosis but poor correlation with disease severity. J. Clin. Gastroenterol..

[6] Lee J, Breslin N, Fallon C, Omorain CJT (2000). Rapid urease tests lack sensitivity in *Helicobacter pylori* diagnosis when peptic ulcer disease presents with bleeding. Am. J. Gastroenterol..

[7] Patel SK, Pratap CB, Verma AK, Jain AK, Dixit VK, Nath GJ (2013). Pseudomonas fluorescens-like bacteria from the stomach: A microbiological and molecular study. World J. Gastroenterol..

[8] Glover B, Teare J, Ashrafian H, Patel N (2020). The endoscopic predictors of *Helicobacter pylori* status: A meta-analysis of diagnostic performance. Ther. Adv. Gastrointest. Endosc..

[9] Ikenoyama Y, Hirasawa T, Ishioka M, Namikawa K, Yoshimizu S, Horiuchi Y, Ishiyama A, Yoshio T, Tsuchida T, Takeuchi Y (2021). Detecting early gastric cancer: Comparison between the diagnostic ability of convolutional neural networks and endoscopists. Dig. Endosc..

[10] Itoh T, Kawahira H, Nakashima H, Yata N (2018). Deep learning analyzes *Helicobacter pylori* infection by upper gastrointestinal endoscopy images. Endosc. Int. Open.

[11] Ueyama H, Kato Y, Akazawa Y, Yatagai N, Komori H, Takeda T, Matsumoto K, Ueda K, Matsumoto K, Hojo M (2021). Application of artificial intelligence using a convolutional neural network for diagnosis of early gastric cancer based on magnifying endoscopy with narrow-band imaging. J. Gastroenterol. Hepatol..

[12] Togashi K (2019). Applications of artificial intelligence to endoscopy practice: The view from Japan Digestive Disease Week. Wiley.

[13] Jia, X. & Meng, M. Q.-H. A deep convolutional neural network for bleeding detection in wireless capsule endoscopy images. in *2016 38th Annual International Conference of the IEEE Engineering in Medicine and Biology Society (EMBC)*, 639–642. 10.1109/EMBC.2016.7590783 28268409

[14] Urban G, Tripathi P, Alkayali T, Mittal M, Jalali F, Karnes W, Baldi P (2018). Deep learning localizes and identifies polyps in real time with 96% accuracy in screening colonoscopy. Gastroenterology.

[15] Zhu Y, Wang Q-C, Xu M-D, Zhang Z, Cheng J, Zhong Y-S, Zhang Y-Q, Chen W-F, Yao L-Q, Zhou P-H (2019). Application of convolutional neural network in the diagnosis of the invasion depth of gastric cancer based on conventional endoscopy. Gastrointest. Endosc..

[16] Nakashima H, Kawahira H, Kawachi H, Sakaki NJ (2018). Artificial intelligence diagnosis of *Helicobacter pylori* infection using blue laser imaging-bright and linked color imaging: A single-center prospective study. Ann. Gastroenterol..

[17] Shichijo S, Nomura S, Aoyama K, Nishikawa Y, Miura M, Shinagawa T, Takiyama H, Tanimoto T, Ishihara S, Matsuo KJ (2017). Application of convolutional neural networks in the diagnosis of *Helicobacter pylori* infection based on endoscopic images. EBioMedicine.

[18] Zheng W, Zhang X, Kim JJ, Zhu X, Ye G, Ye B, Wang J, Luo S, Li J, Yu TJC (2019). High accuracy of convolutional neural network for evaluation of *Helicobacter pylori* infection based on endoscopic images: Preliminary experience. Clin. Transl. Gastroenterol..

[19] Liu G, Hua J, Wu Z, Meng T, Sun M, Huang P, He X, Sun W, Li X, Chen Y (2020). Automatic classification of esophageal lesions in endoscopic images using a convolutional neural network. Ann. Transl. Med..

[20] Sobri Z, Sakim HAM (2012). Texture color fusion based features extraction for endoscopic gastritis images classification. Int. J. Comput. Electr. Eng..

[21] Jain S, Seal A, Ojha A, Yazidi A, Bures J, Tacheci I, Krejcar O (2021). A deep CNN model for anomaly detection and localization in wireless capsule endoscopy images. Comput. Biol. Med..

[22] Zhang C, Wu J, Chen Z, Liu W, Li M, Jiang S (2021). Dense-CNN: Dense convolutional neural network for stereo matching using multiscale feature connection. Signal Process. Image Commun..

[23] Hsu P-I, Tsai F-W, Kao S-S, Hsu W-H, Cheng J-S, Peng N-J, Tsai K-W, Hu H-M, Wang Y-K, Chuah S-KJ (2017). Ten-day quadruple therapy comprising proton pump inhibitor, bismuth, tetracycline, and levofloxacin is more effective than standard levofloxacin triple therapy in the second-line treatment of *Helicobacter pylor*i infection: A randomized controlled trial. J. Am. College Gastroenterol..

[24] Wu J (2017). Introduction to convolutional neural networks. Natl. Key Lab. Novel Softw. Technol..

[25] Kim P (2017). Convolutional Neural Network.

[26] Radzi, S. A. & Khalil-Hani, M. Character recognition of license plate number using convolutional neural network. in *International Visual Informatics Conference*, 45–55.

[27] Albawi, S., Mohammed, T. A. & Al-Zawi, S. Understanding of a convolutional neural network. in *2017 International Conference on Engineering and Technology (ICET)*, 1–6.

[28] Roy, A. G., Navab, N. & Wachinger, C. Concurrent spatial and channel ‘squeeze & excitation in fully convolutional networks. in *International Conference on Medical Image Computing and Computer-Assisted Intervention*, 421–429.

[29] Li X, Su H, Liu G (2020). Insulator defect recognition based on global detection and local segmentation. IEEE Access.

[30] Liu, Z., Wang, H., Lei, W. & Wang, G. CSAF-CNN: Cross-layer spatial attention map fusion network for organ-at-risk segmentation in head and neck CT images. in *2020 IEEE 17th International Symposium on Biomedical Imaging (ISBI)*, 1522–1525.

[31] Yan H, Chen A (2021). A novel improved brain tumor segmentation method using deep learning network. J. Phys. Conf. Ser..

[32] Roy AG, Navab N, Wachinger C (2018). Recalibrating fully convolutional networks with spatial and channel “squeeze and excitation” blocks. IEEE Trans. Med. Imaging.

[33] Guillaumin, M., Verbeek, J. & Schmid, C. Multimodal semi-supervised learning for image classification. In *2010 IEEE Computer Society Conference on Computer Vision and Pattern Recognition*, 902–909.

[34] Learned-Miller EG (2014). Introduction to Supervised Learning.

[35] Murray RF (2011). Classification images: A review. J. Vis..

[36] Chen N, Blostein D (2007). A survey of document image classification: problem statement, classifier architecture and performance evaluation. IJDAR.

[37] Lever J (2016). Classification evaluation: It is important to understand both what a classification metric expresses and what it hides. Nat. Methods.

[38] Fix E (1985). Discriminatory Analysis: Nonparametric Discrimination, Consistency Properties.

[39] Cortes C, Vapnik V (1995). Support-vector networks. Mach. Learn..

[40] Freund Y, Schapire RE (1997). A decision-theoretic generalization of on-line learning and an application to boosting. J. Comput. Syst. Sci..

[41] Ho, T. K. Random decision forests. in *Proceedings of 3rd International Conference on Document Analysis and Recognition*, 278–282.

[42] Friedman JH (2001). Greedy function approximation: a gradient boosting machine. Ann. Stat..

[43] Chen, T. & Guestrin, C. Xgboost: A scalable tree boosting system. in *Proceedings of the 22nd ACM Sigkdd International Conference on Knowledge Discovery and Data Mining*, 785–794.

[44] Ke G, Meng Q, Finley T, Wang T, Chen W, Ma W, Ye Q, Liu T-Y (2017). Lightgbm: A highly efficient gradient boosting decision tree. Adv. Neural. Inf. Process. Syst..

[45] Prokhorenkova, L., Gusev, G., Vorobev, A., Dorogush, A.V. & Gulin, A. *CatBoost: Unbiased Boosting with Categorical Features* (2017). arXiv:1706.09516.

